# Are Probiotics or Prebiotics Useful in Pediatric Irritable Bowel Syndrome or Inflammatory Bowel Disease?

**DOI:** 10.3389/fmed.2014.00023

**Published:** 2014-08-28

**Authors:** Stefano Guandalini

**Affiliations:** ^1^Section of Gastroenterology, Hepatology and Nutrition, Department of Pediatrics, The University of Chicago, Chicago, IL, USA

**Keywords:** irritable bowel syndrome, functional gastrointestinal disorders, inflammatory bowel disease, probiotics, VSL#3, *Lactobacillus*, Crohn’s disease, ulcerative colitis

## Abstract

Treatment options for irritable bowel syndrome (IBS) and inflammatory bowel disease (IBD) are notoriously either inadequate (IBS) or loaded with potentially serious side effects and risks (IBD). In recent years, a growing interest in effective and safer alternatives has focused on the potential role of probiotics and their metabolic substrates, prebiotics. It is in fact conceivable that the microbiome might be targeted by providing the metabolic fuel needed for the growth and expansion of beneficial microorganisms (prebiotics) or by administering to the host such microorganisms (probiotics). This review presents a concise update on currently available data, with a special emphasis on children. Data for prebiotics in IBS are scarce. Low doses have shown a beneficial effect, while high doses are counterproductive. On the contrary, several controlled trials of probiotics have yielded encouraging results. A meta-analysis including nine randomized clinical trials in children showed an improvement in abdominal pain for *Lactobacillus* GG, *Lactobacillus reuteri* DSM 17938, and the probiotic mixture VSL#3. The patients most benefiting from probiotics were those with predominant diarrhea or with a post-infectious IBS. In IBD, the use of prebiotics has been tested only rarely and in small scale clinical trials, with mixed results. As for probiotics, data in humans from about three dozens clinical trials offer mixed outcomes. So far, none of the tested probiotics has proven successful in Crohn’s disease, while in ulcerative colitis a recent meta-analysis on 12 clinical trials (1 of them in children) showed efficacy for the probiotic mixture VSL#3 in contributing to induce and to maintain remission. It is evident that this is a rapidly evolving and promising field; more data are very likely to yield a better understanding on what strains should be used in different specific clinical settings and in what doses.

## Introduction

Irritable bowel syndrome (IBS) is a very common disorder, affecting up to 20% of children and teenagers (1), equally involving boys and girls (2) and causing an important health and social burden and with substantial costs (3, 4). IBS is a functional gastrointestinal disorder (FGID) characterized by the association of recurrent abdominal pain to a change in stool consistency or frequency toward a diarrhea- or a constipation-predominant pattern. Its treatment remains largely unsatisfactory.

Inflammatory bowel diseases (IBDs) are a group of chronic, incurable inflammatory disorders of the gastrointestinal tract [mostly represented by Crohn’s disease (CD) and ulcerative colitis]. Their cause is unknown, but genetic, immunologic, and environmental factors are involved. The peak age of onset for IBD is 15–30 years, but it may occur at any age. About 10% of cases have their onset before the age of 18, with CD being more frequent in girls, while ulcerative colitis is more common in boys (5). The disease is one of the most prevalent gastrointestinal disease burdens in Western societies and it has become more widespread; its rise in incidence has been reported in all age groups including early childhood (6).

Prebiotics (7) are carbohydrates that transit undigested through the small intestine and eventually reach the colon where they stimulate the growth and/or the activity of beneficial bacteria, mostly bifidobacteria and lactic acid bacteria. Prebiotics include galacto-oligosaccharides (GOS), fructo-oligosaccharides (FOS), inulin, and β-glucan (8). The potential for their use in IBS and IBD, in addition to their stimulatory effect on potential probiotic microorganisms, stems also from the observation that some prebiotics (e.g., mannanoligosaccharides, inulin, or β-glucan), are thought to be “immunosaccharides,” i.e., they might act as direct stimulators of innate immunity (9).

Probiotics on the other hand are defined as “live microorganisms that, when administered in adequate amounts, confer a health benefit on the host” (10).

There is a conceivable benefit in their use in both, IBS and IBD. In IBS, alterations in the gut microbiota composition have been well described (reviewed in a recent report by the Rome working team) (11) and recent evidence has elucidated the growing importance of the microbiota–gut–brain axis in the development of IBS (12). As for IBD, the potential role for such compounds appears logical: the endogenous intestinal microbiota plays a central role in the disease development, and various probiotics have been found effective in a variety of animal models.

This review will examine the current status of the utilization of probiotics and prebiotics in children for both disorders.

## Probiotics in IBS

Irritable bowel syndrome is defined (13) by the association of abdominal pain with a change in stool pattern in the absence of an organic cause. It is a very prevalent FGID, affecting up to 50% of the children and teenagers seen by a gastroenterology practice (14, 15) and causing significant health care costs and a negative impact on quality of life (16).

Changes in gastrointestinal motility, brain/gut interaction, low-grade inflammation, psychosocial disturbance, visceral hypersensitivity, and altered microbiota appear to contribute to various extents to its onset (17, 18). It is also possible that IBS results from different pathogenetic factors that contribute in various degrees in different patients (19).

How could probiotics be useful in IBS? IBS appears often to follow an episode of gastroenteritis (“post-infectious IBS”), (20) and intestinal microbiota changes have been described in patients with IBS (21–23); furthermore, it is quite conceivable that microbiota may interfere with additional factors involved in the pathophysiology of IBS (24, 25).

In adults, several trials have been published, mostly of a small scale and bearing in general promising results (17, 26, 27).

In children, however, fewer randomized clinical trials (RCTs) are available.

### Effects of probiotics on abdominal pain

We conducted a double-blinded, placebo-controlled, cross-over RCT (28) examining the effect of the probiotic mixture VSL#3, a proprietary preparation consisting of a high concentration of eight different probiotic strains (*Lactobacillus acidophilus*, *Lactobacillus plantarum*, *Lactobacillus casei*, *Lactobacillus bulgaricus*, *Bifidobacterium breve*, *Bifidobacterium longum*, *Bifidobacterium infantis*, and *Streptococcus thermophilus*), in children and teenagers with IBS. Our results showed that while the patients were taking VSL#3, they experienced a significant (*p* < 0.05) improvement in the global subjective relief of symptoms score as well as in the score for abdominal pain/discomfort. Similarly, significant benefits were found for bloating/gassiness and for caregivers’ satisfaction with their child improvement, while no significant improvement was seen for diarrhea or constipation.

In 2014, systematic review and meta-analysis (29) on the effect of different probiotics as a treatment for FGID in children and teenagers included nine trials, five of whom, including our trial (28), focused on abdominal pain-related FGID (15, 28, 30–32), and four on bowel changes-related FGID (33–36).

The meta-analysis concluded that the use of *Lactobacillus* GG, *Lactobacillus reuteri* DSM 17938 and VSL#3 significantly increased treatment success in children with abdominal pain-related FGID, particularly those with associated bowel changes. Of interest, *L. reuteri* DSM 17938’s significant decrease of abdominal pain intensity persisted after the removal of the probiotic, indicating a lasting effect of the supplementation (31).

### Effects of probiotics on functional diarrhea or constipation

Notably, the two RCTs that assessed the effect of probiotics (*Lactobacillus* GG and VSL#3) on the parameter of diarrhea (15, 28) did not show any benefit of the intervention.

Four studies included in the meta-analysis assessed constipation-related FGID. The probiotics used were *Lactobacillus* GG associated with lactulose, *L. casei rhamnosus* Lcr35, *B. longum*, and *Bifidobacterium lactis* DN-173 010 (33–36). The results were disappointing, with no evidence that probiotics are more effective than placebo in overall outcome of treatment or in increasing defecation frequency in constipated children and with inconsistent data on the improvement in stool consistency (34, 35).

## Probiotics in IBD

It is now well established that intestinal microbiota play an important role in IBD (37). Probiotics appear therefore as a logical option to positively influence the clinical course of IBD. They possess anti-inflammatory action and enhance the gut barrier, and since the inflammation in IBD results from an altered mucosal immune response to luminal bacterial antigens, the use of live microorganisms may conceivably favorably affect the overall microbial balance.

The mechanisms of action underlying the benefits of probiotics in IBD have been largely and quite extensively investigated in a variety of *in vitro* and *in vivo* animal experiments [reviewed in Ref. (38)]. It is thought that in general, the effects manifested by a specific probiotic depend on its metabolic properties, the molecules surface-expressed molecules, and the products they secrete (39).

However, in spite of the strength of the conceptual basis for the beneficial use of probiotics, and of the extensive experimental data in animal models, the existing literature – and in particularly RCTs – on the use of probiotics in IBD is relatively limited. I will consider here only published studies reporting RCTs with a comparator, either placebo or an accepted standard therapy.

### Crohn’s disease

A meta-analysis on probiotics in adult IBD published in 2012 (40) included eight papers in CD: four conducted in active CD (only one of which placebo-controlled). The latter trial reported a lower relapse rate after 12 months receiving of *Escherichia coli* Nissle 1917 added to prednisolone therapy (41). The other four studies were conducted in patients with CD in remission: two used *Lactobacillus* GG (42, 43) and two *Lactobacillus johnsonii* (44, 45). In none of the studies were probiotics more effective than placebo in preventing relapses.

A subsequent and recent meta-analysis (46) included 23 RCT (12 in UC, 4 in “pouchitis,” and 7 in CD, only 3 of them in children) with a total of 1763 participants: again results were quite disappointing, as in none of the trials in CD probiotics appeared to be of any significant benefit. Focusing on the three pediatric trials (41, 43, 47) where the remission/response rates in acute CD were reported, in none was any positive effect of probiotics supplementation shown (*p* = 0.35, RR = 0.89).

Seven RCTs (including only one in children, reported in more detail below) examined the frequency of and timing to clinical relapses in CD patients who were in remission. Again, no difference was found between the probiotics-supplemented patients and the control ones (*p* = 0.71, RR = 1.09).

In children, a randomized, placebo-controlled trial of the probiotic *Lactobacillus* GG (48) with the aim of comparing the remission in patients that receive *Lactobacillus* GG added to standard therapy was unable to demonstrate that this probiotic would prolong remission time in patients with CD already in remission on standard therapy. In fact, the time to relapse and proportion of patients relapsing was essentially identical in both *Lactobacillus* GG and placebo groups.

### Ulcerative colitis

Twelve of the 23 RCTs included in the 2014 meta-analysis (46) were conducted in UC patients.

Nine studies analyzed the remission/response in acute UC, utilizing bifidobacteria, *E. coli* Nissle 1917, and VSL#3, while the overall analysis of these RCTs showed significant benefit from the use of probiotics, the sub-analysis based on probiotic utilized documented such significant effect only for VSL#3 (49–53).

*Escherichia coli* Nissle 1917 is one of the few probiotics licensed as a medicine in Europe where it is utilized in patients with ulcerative colitis; its genome has recently been characterized (54). In spite of a promising open-label pilot trial in 34 children in 2008 (55), no further trial have been conducted in children. In adults, the meta-analysis’ subgroup analysis (46) failed to document evidence of efficacy, and a recent trial in 100 patients (56) not included in the meta-analysis, also failed to show any benefit in addition to conventional therapy. In addition, treatment with this probiotic without a previous antibiotic treatment appeared to be actually detrimental, with fewer patients reaching clinical remission (56).

Are probiotics efficacious in maintaining the remission achieved pharmacologically? Five trials in UC addressed this issue, showing no advantage of probiotics administration when compared to placebo only (RR 0.89, 95% CI 0.66–1.21). It is, however, remarkable that the only pediatric trial conducted with VSL#3 (49) showed actually efficacy of the probiotic (RR 0.29, 95% CI 0.10–0.83). This trial included 29 children, with a recent diagnosis of mild to moderate UC who received initially prednisone and mesalazine plus either placebo or VSL#3 for induction, and once remission had been obtained, they remained on mesalazine plus either placebo or VSL#3. The patients were evaluated at 1 month, 2 months, 6 months, and 1 year after diagnosis or at the time of relapse. An endoscopy was additionally performed at the time of diagnosis and then repeated at 6 months, 12 months, or at the time of relapse. The study found that not only the remission but also the relapse rates were better in the patients treated with VSL#3; furthermore, endoscopic and histological scores were significantly better in the VSL#3 group than in the placebo group (*p* < 0.05).

Another pediatric trial, not included in the 2014 meta-analysis, examined 31 children with mild to moderate ulcerative proctitis/proctosigmoiditis (57); they received by enema a solution with *L. reuteri* ATCC 55730 or placebo during 8 weeks, in addition to the oral treatment with mesalazine. A clinical, endoscopic, histological, and immune evaluation (IL-10, IL-1b, TNFα, and IL-8) was performed. Clinical and endoscopic improvements were shown to be better in the probiotic group, and the histological score was significantly decreased in the *L. reuteri* group (*p* < 0.01). In the *L. reuteri* group, a significant increase in the mucosal expression levels of IL-10 and a significant decrease in the levels of IL-1b, TNFα, and IL-8 mucosal expression levels (*p* < 0.01) were also documented.

## Conclusions on the Efficacy of Probiotics in Pediatric IBS and IBD

In IBS, some strains of probiotics offer a modest but meaningful benefit in the treatment of FGID in children, and especially in the treatment of abdominal pain. Considering their outstanding safety profile and the scarcity of alternative safe treatment options, they appear to have an interesting role.

As for IBD, at present there is no evidence of efficacy for any strain in CD. It must, however, be considered that CD is a very heterogeneous condition, with varying degrees of genetic components, very different phenotypes, and variable degrees of severity. It is therefore very likely that the patient-probiotic profiles so far examined are not exhaustive; a better refinement of the strains, their dosage, and their specific applications to well-identified subgroups of patients may yield valuable information and possibly some useful therapeutic implications. In ulcerative colitis on the other hand, there is already evidence of efficacy for the probiotic mixture VSL#3. Further, larger prospective RCTs appear definitely warranted.

## Prebiotics in IBS

Four RCTs have investigated the efficacy of prebiotics to treat IBS symptoms, all of them in adult patients. The first RCT was a cross-over trial where 21 patients with IBS were given 6 g/day of FOS (58). There were no significant deviations in symptoms for the treated or control group. The second study conducted by Olesen and Gudmand-Hoyer involved 96 patients with IBS who were given either 20 g/day of FOS or placebo for 12 weeks (59). This high dose increased symptoms at 4–6 weeks thereby suggesting high dosage of prebiotics such as FOS would be ineffective and potentially even dangerous for treatment of IBS. The third study involved 105 patients with functional bowel disorders who were administered 5 g/day of oligofructose (60). Although improvements in global symptoms and bloating were noted, only 50 patients could be included in the final analysis due to withdrawal or violation of protocol. The latest study was conducted over a period of 4 weeks and involved random administration of 3.5 g/day of GOS, 7 g/day of GOS, or placebo to 44 IBS patients (61). The administration of GOS at both doses resulted in an increased predominance of bifidobacteria in fecal samples; and the lowest dose also resulted in significant improvement in bloating, flatulence, and global relief of IBS symptoms. However, higher dosage of GOS caused worse bloating symptoms.

Thus, with all the risks involved in drawing conclusions from only four studies and keeping in mind no data are available so far in children, it is quite conceivable a low to moderate dose of prebiotics might provide some symptom relief, while higher doses may end up being detrimental. This negative effect may well be related to increased luminal gas production following fermentation. In fact, it has been shown that a diet high in fermentable carbohydrates elevated hydrogen gas in 15 patients with IBS and 15 health control patients (62). This resulted in increased abdominal pain, bloating, and flatulence among IBS patients as well as flatulence among healthy controls. To further support this concept, it has recently been shown that a diet low in fermentable oligosaccharides, disaccharides, monosaccharides, and polyols (FODMAP) is beneficial in improving IBS symptoms (63), while a diet rich in these carbohydrates increases fasting colonic volume and breath hydrogen in healthy volunteers (64).

## Prebiotics in IBD

In spite of a substantial amount of promising data obtained in animal systems (65–68) there are few studies investigating the efficacy of prebiotics in humans with IBD, and none in children.

An open-label study with FOS supplementation in 10 adult CD patients (69) showed that FOS improved disease activity index, increased fecal bifidobacteria concentrations and modified mucosal dendritic cell function.

A more recent RCT in 103 patients with CD randomized to receive FOS (*n* = 54) or placebo (*n* = 49) failed to show any significant difference in the number of patients achieving a clinical response between the FOS and placebo groups (70).

In an open trial involving patients with mild to moderate UC who had been unresponsive or intolerant to standard treatment, patients received 20–30 mg of germinated barley food (GBF) in a daily feeding (71). At 4 weeks, the GBF treatment had resulted in clinical and endoscopic improvement regardless of initial extent of disease, and the improvement was linked to elevated butyrate concentration in stool. A study by Kanauchi et al. (72) found that GBF increased levels of *Bifidobacterium* and *Eubacterium limosum* and appeared to prolong remission in UC patients.

Casellas et al. in a placebo-controlled RCT on 19 UC patients treated with mesalazine showed that the group who received oligofructose-enriched inulin supplementation had a lower fecal calprotectin than controls (73).

## Conclusions on the Efficacy of Prebiotics in Pediatric IBS and IBD

Prebiotics in IBS have not been studied in children; hence no conclusion can obviously be drawn. The tricky dose–effect relationship found in adult makes their use in children not promising and probably potentially quite limited.

In IBD, again no data are available in pediatric age. Here, however, considering some positive effect in the limited studies available in adults, it would appear that prebiotics may have some potential. Needless to say, much more evidence needs to be generated before any meaningful clinical indication can be identified.

## Conflict of Interest Statement

The author declares that the research was conducted in the absence of any commercial or financial relationships that could be construed as a potential conflict of interest.
